# Supplementary material for: Long-Term Effects of Ambient Particulate and Gaseous Pollutants on Serum High-Sensitivity C-Reactive Protein Levels: A Cross-Sectional Study Using KoGES-HEXA Data
Source: Int J Environ Res Public Health. 2022 Sep 14;19(18):11585. doi: 10.3390/ijerph191811585 (PMC9517608; doi:10.3390/ijerph191811585)
Supplement: Supplementary file 1 [file ijerph-19-11585-s001.zip › ijerph-1836753-supplementary.pdf]

Figure S1. Spatial distribution of the geocoded residential addresses and the long-term air pollutant concentrations of the 60,581 participants in Korea.
(a) Particulate matters

**PM_{10}**	**$PM_{2.5}$**

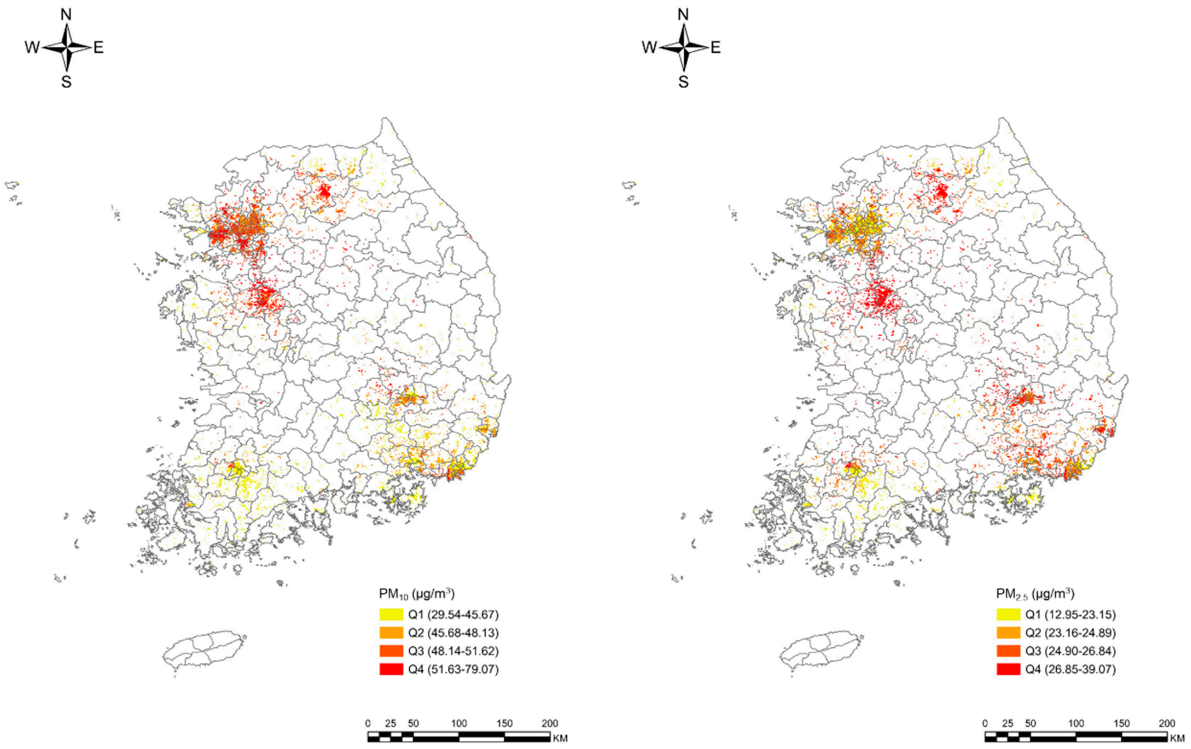

Particulate matter with aerodynamic diameter < 10 μm (PM_{10}) and < 2.5 μm ($PM_{2.5}$) were measured in every 1 km-grid resolution. Dots are the locations of the observations, and colors represent the quartiles of each pollutant.

Figure S1. *Cont.*
(b) Gaseous pollutants

SO_2	NO_2	O_3

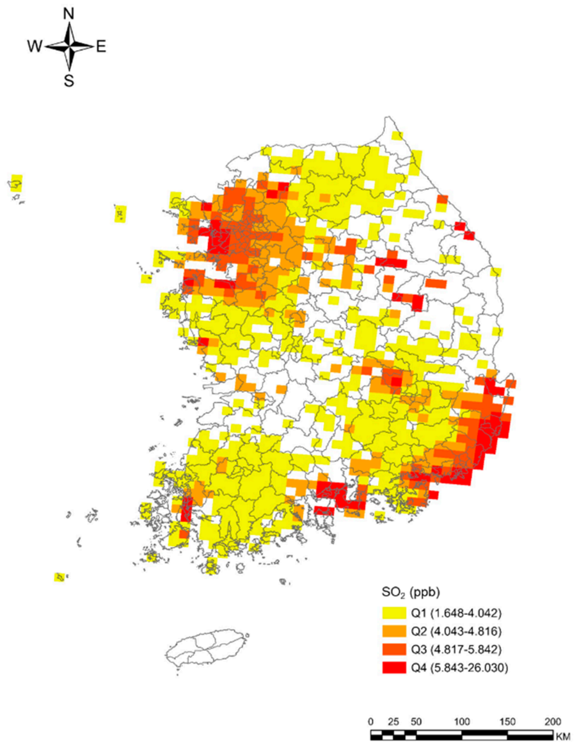

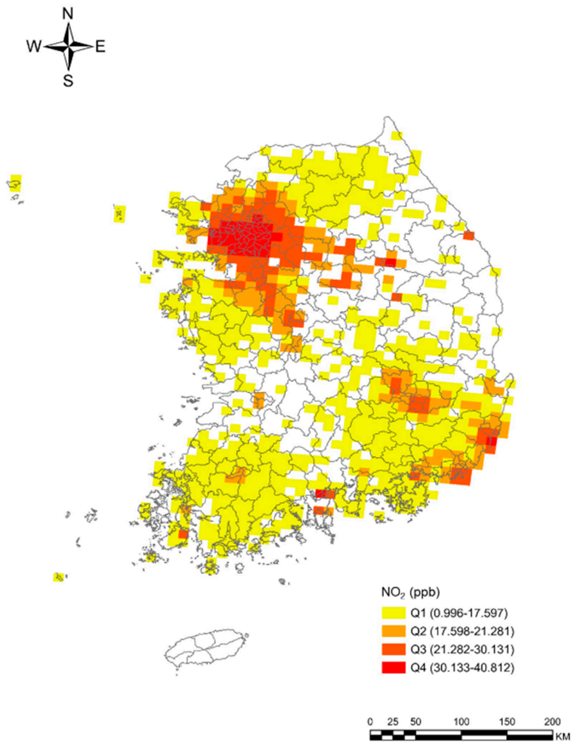

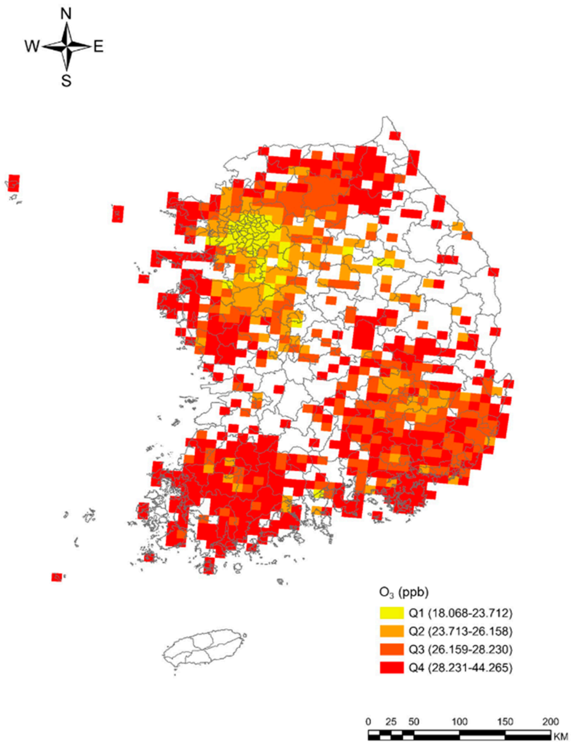

Gaseous materials including sulfur dioxide (SO_2), nitrogen dioxide (NO_2), and ozone (O_3) were measured in every 9 km-grid resolution.
Dots are the locations of the observations, and colors represent the quartiles of each pollutant.

Table S1. Configurations for the meteorology, emission, and chemical transport model used in this study.

**Models**	**Types and versions of the models**
Meteorology	WRF (Weather Research and Forecasting) version 3.6.1
Emission	SMOKE (Sparse Matrix Operator Kernel Emissions) version 2.7
	Emission inventories: CAPSS (Clean Air Policy Support System) for Korea, MEIC (Multi-resolution Emission Inventory for China) for China, and REAS (Regional Emission inventory in Asia) for East Asian countries other than Korea and China.
Chemical transport	US EPA Model-3 CMAQ (Community Multiscale Air Quality) version 4.7.1
	Spatial coverage: outer coarse domain with 27 km resolution for East Asia and finer domain with 9 km resolution for Korea

Table S2. Summary of the model performance evaluation (2010–2017).

Pollutant (unit)	Year[a]	R	R^2	RMSE	IOA
PM_{10} (μg/m^3)	2010	0.87	0.75	16.71	0.91
	2011	0.91	0.82	14.83	0.94
	2012	0.86	0.75	11.59	0.92
	2013	0.92	0.85	10.46	0.95
	2014	0.89	0.78	13.32	0.92
	2015	0.84	0.71	17.54	0.90
	2016	0.84	0.71	13.01	0.89
	2017	0.86	0.74	12.10	0.91
	Average	0.87	0.76	13.70	0.92
$PM_{2.5}$ (μg/m^3)	2015	0.77	0.60	9.25	0.87
	2016	0.81	0.66	7.78	0.88
	2017	0.85	0.72	7.91	0.90
	Average	0.81	0.66	8.31	0.88
SO_2 (ppm)	2010	0.70	0.49	0.0029	0.82
	2011	0.62	0.38	0.0034	0.76
	2012	0.69	0.47	0.0029	0.82
	2013	0.71	0.51	0.0027	0.84
	2014	0.65	0.42	0.0030	0.79
	2015	0.66	0.44	0.0027	0.80
	2016	0.56	0.31	0.0030	0.72
	2017	0.59	0.35	0.0028	0.74
	Average	0.65	0.42	0.0029	0.79
NO_2 (ppm)	2010	0.88	0.77	0.0066	0.94
	2011	0.89	0.78	0.0065	0.94
	2012	0.88	0.77	0.0063	0.93
	2013	0.88	0.77	0.0066	0.94
	2014	0.87	0.75	0.0068	0.93
	2015	0.84	0.71	0.0073	0.92
	2016	0.80	0.64	0.0077	0.88
	2017	0.85	0.72	0.0064	0.92
	Average	0.86	0.74	0.0068	0.93
O_3 (ppm)	2010	0.75	0.56	0.0079	0.84
	2011	0.77	0.59	0.0078	0.86
	2012	0.80	0.64	0.0074	0.88
	2013	0.91	0.84	0.0052	0.95
	2014	0.81	0.66	0.0079	0.89
	2015	0.85	0.72	0.0071	0.91
	2016	0.82	0.66	0.0077	0.89
	2017	0.79	0.62	0.0085	0.87
	Average	0.81	0.66	0.0074	0.89

PM_{10}: particulate matter with aerodynamic diameter < 10 μm; $PM_{2.5}$: particulate matter with aerodynamic diameter < 2.5 μm; SO_2: sulfur dioxide; NO_2: nitrogen dioxide; O_3: ozone; R: correlation coefficient; R^2: square of the correlation coefficient; RMSE: root mean square error; IOA: index of agreement.

[a] The air quality monitoring data for $PM_{2.5}$ was available from 2015.

Table S3. Sensitivity analysis on the models with candidate covariates for the associations between hs-CRP levels and long-term exposure to air pollutants.

Pollutant	Model	% change (95% CI)[a]	OR (95% CI)[b]
PM_{10}	Model 1[c]	4.643 (3.526, 5.773)	1.073 (1.014, 1.135)
	Model 2[d]	3.933 (2.866, 5.012)	1.067 (1.009, 1.130)
	Model 3 (Main model)[e]	3.747 (2.682, 4.823)	1.065 (1.007, 1.128)
$PM_{2.5}$	Model 1[c]	4.239 (3.112, 5.379)	1.099 (1.038, 1.163)
	Model 2[d]	2.858 (1.786, 3.941)	1.080 (1.020, 1.143)
	Model 3 (Main model)[e]	3.683 (2.568, 4.811)	1.076 (1.016, 1.139)
SO_2	Model 1[c]	2.421 (1.704, 3.143)	1.058 (1.020, 1.097)
	Model 2[d]	1.890 (1.203, 2.583)	1.047 (1.009, 1.086)
	Model 3 (Main model)[e]	1.787 (1.101, 2.478)	1.045 (1.007, 1.084)
NO_2	Model 1[c]	2.450 (1.219, 3.695)	1.003 (0.941, 1.069)
	Model 2[d]	3.486 (2.286, 4.700)	1.025 (0.961, 1.094)
	Model 3 (Main model)[e]	3.312 (2.115, 4.524)	1.025 (0.961, 1.094)
O_3	Model 1[c]	−3.366 (−4.559, −2.159)	0.978 (0.916, 1.044)
	Model 2[d]	−3.975 (−5.120, −2.815)	0.960 (0.899, 1.026)
	Model 3 (Main model)[e]	−3.812 (−4.958, −2.651)	0.961 (0.899, 1.027)

PM_{10}, $PM_{2.5}$: particulate matter with aerodynamic diameter < 10 μm and < 2.5 μm, respectively; SO_2: sulfur dioxide; NO_2: nitrogen dioxide; O_3: ozone; Hs-CRP: high sensitivity C-reactive protein.

[a] Estimates are presented as percentage changes with 95% confidence intervals in hs-CRP levels per 1 unit increase in 2-year average ambient air pollution exposure (corresponding IQR of each pollutant: 5.95 $\mu g/m^3$ for PM_{10}, 3.70 $\mu g/m^3$ for $PM_{2.5}$, 1.80 ppb for SO_2, 12.54 ppb for NO_2, and 4.52 ppb for O_3).

[b] Estimates are presented as odds ratios and 95% confidence intervals of low-grade inflammation (hs-CRP levels > 3 mg/L) per 1 unit increase in 2-year average ambient air pollution exposure (corresponding IQR of each pollutant: 5.95 $\mu g/m^3$ for PM_{10}, 3.70 $\mu g/m^3$ for $PM_{2.5}$, 1.80 ppb for SO_2, 12.54 ppb for NO_2, and 4.52 ppb for O_3).

[c] Adjusted for age, sex, residential area, time (year, season, and weekday of examination), and meteorological factors (2-day moving average of temperature and relative humidity).

[d] Adjusted for the covariates in model 1 as well as for body mass index, smoking status, alcohol consumption, regular exercise, occupation, education, and marital status.

[e] Adjusted for the covariates in model 2 as well as for medical history of diseases (diabetes, hypertension, dyslipidemia, cardio- and cerebrovascular disease, and cancer).

Table S4. Effect modifications of associations between hs-CRP levels and long-term air pollutant concentrations by participant characteristics.

	% change (95% CI)				
	PM_{10}	$PM_{2.5}$	SO_2	NO_2	O_3
**All** (n=60,581)	3.747 (2.682, 4.823)	3.683 (2.568, 4.811)	1.787(1.101, 2.478)	3.312 (2.115, 4.524)	−3.812 (−4.958, −2.651)
**Age**					
41−64 years (n=44,856)	3.502 (2.278, 4.741)	1.989 (0.768, 3.224)	0.926 (0.159, 1.698)	3.145 (1.793, 4.515)	−3.911 (−5.209, −2.595)
≥65 years (n=15,725)	4.357 (2.202, 6.558)	4.571 (2.355, 6.834)	2.838 (1.350, 4.348)	3.347 (0.800, 5.958)	−3.841 (−6.261, −1.359)
p-interaction	0.215	0.012	0.154	0.807	0.934
**Sex**					
Males (n=20,282)	2.583 (0.828, 4.369)	2.908 (1.112, 4.736)	2.643 (1.343, 3.960)	2.219 (0.117, 4.366)	−1.836 (−3.859, 0.229)
Females (n=40,299)	4.821 (3.474, 6.185)	2.899 (1.560, 4.256)	1.543 (0.736, 2.356)	4.147 (2.682, 5.633)	−5.202 (−6.592, −3.791)
p-interaction	0.025	0.919	0.203	0.040	0.003
**Body mass index (BMI)**					
<25 kg/m^2 (n=40,993)	3.222 (1.915, 4.546)	2.430 (1.102, 3.777)	1.741 (0.895, 2.594)	2.877 (1.417, 4.357)	−3.209 (−4.623, −1.775)
≥25 kg/m^2 (n=19,588)	5.911 (4.002, 7.855)	4.640 (2.762, 6.551)	2.256 (1.040, 3.487)	3.798 (1.642, 6.001)	−5.076 (−7.094, −3.013)
p-interaction	0.003	0.065	0.094	0.205	0.331
**Smoking status**					
Non-smoker (n=45,647)	4.311 (3.066, 5.571)	2.445 (1.204, 3.701)	1.521 (0.752, 2.296)	3.942 (2.570, 5.334)	−4.754 (−6.059, −3.430)
Ex-smoker (n=10,252)	0.880 (−1.589, 3.410)	2.939 (0.351, 5.594)	1.233 (−0.578, 3.077)	−0.145 (−3.062, 2.860)	−0.270 (−3.206, 2.754)
Current smoker (n=4682)	5.096 (1.410, 8.916)	4.186 (0.559, 7.944)	5.744 (3.024, 8.535)	4.942 (0.453, 9.632)	−2.920 (−7.010, 1.351)
p-interaction	0.168	0.822	0.011	0.060	0.105
**Regular exercise**					
Yes (n=35,617)	2.992 (1.533, 4.473)	2.532 (1.105, 3.980)	0.990 (0.131, 1.855)	2.172 (0.605, 3.764)	−2.983 (−4.518, −1.424)
No (n=24,964)	4.523 (2.955, 6.114)	3.032 (1.417, 4.672)	3.115 (1.972, 4.270)	4.600 (2.746, 6.487)	−4.707 (−6.439, −2.944)
p-interaction	0.232	0.655	0.002	0.034	0.330
**Diabetes**					
Yes (n=8161)	2.624 (−0.324, 5.659)	3.256 (0.249, 6.353)	1.477 (−0.447, 3.438)	2.911 (−0.595, 6.541)	−2.641 (−6.024, 0.863)
No (n=52,420)	4.032 (2.892, 5.185)	2.777 (1.634, 3.934)	1.831 (1.098, 2.569)	3.367 (2.097, 4.654)	−4.013 (−5.227, −2.783)
p-interaction	0.591	0.611	0.855	0.891	0.705
**Hypertension**					
Yes (n=20,982)	4.794 (2.939, 6.681)	4.461 (2.608, 6.347)	2.149 (0.954, 3.359)	2.659 (0.576, 4.785)	−4.336 (−6.338, −2.291)
No (n=39,599)	3.321 (2.023, 4.636)	1.815 (0.508, 3.140)	1.622 (0.785, 2.465)	3.724 (2.263, 5.205)	−3.644 (−5.036, −2.231)
p-interaction	0.059	0.015	0.242	0.736	0.465
**Dyslipidemia**					
Yes (n=25,092)	4.661 (3.014, 6.334)	3.500 (1.851, 5.176)	1.912 (0.864, 2.971)	3.493 (1.652, 5.367)	−4.371 (−6.130, −2.579)
No (n=35,489)	3.237 (1.845, 4.647)	2.193 (0.794, 3.611)	1.687 (0.782, 2.600)	3.292 (1.721, 4.886)	−3.573 (−5.075, −2.047)
p-interaction	0.096	0.352	0.547	0.434	0.498
**Cardio- and cerebrovascular disease**					
Yes (n=3390)	6.508 (1.822, 11.408)	2.753 (−1.958, 7.691)	7.280 (3.946, 10.720)	9.042 (3.126, 15.297)	−6.985 (−12.042, −1.637)
No (n=57,191)	3.595 (2.503, 4.699)	2.736 (1.639, 3.846)	1.523 (0.822, 2.229)	3.044 (1.823, 4.280)	−3.642 (−4.818, −2.453)
p-interaction	0.257	0.775	<0.001	0.009	0.213

PM_{10}: particulate matter with aerodynamic diameter < 10 μm; $PM_{2.5}$: particulate matter with aerodynamic diameter < 2.5 μm; SO_2: sulfur dioxide; NO_2: nitrogen dioxide; O_3: ozone; Hs-CRP: high sensitivity C-reactive protein.

Estimates are presented as percentage changes with 95% confidence intervals in hs-CRP levels per IQR increase in 2-year average ambient air pollution exposure (corresponding IQR of each pollutant: 5.95 μg/m^3 for PM_{10}, 3.70 μg/m^3 for $PM_{2.5}$, 1.80 ppb for SO_2, 12.54 ppb for NO_2, and 4.52 ppb for O_3). Adjusted for age, sex, BMI, smoking status, drinking status, regular exercise, occupation, education, marital status, residential area, year, season, and weekday of examination, medical history of diseases (diabetes, hypertension, dyslipidemia, cardio- and cerebrovascular disease, and cancer), and meteorological factors (2-day moving average of temperature and relative humidity). Age, sex, BMI, smoking status, regular exercise, and medical histories were not adjusted in the stratified analysis.

Table S5. Effect modifications of associations between systemic low-grade inflammation and long-term air pollutant concentrations by participant characteristics.

	OR (95% CI)				
	PM_{10}	$PM_{2.5}$	SO_2	NO_2	O_3
**All** (n=60,581)	1.065 (1.007, 1.128)	1.076 (1.016, 1.139)	1.045 (1.007, 1.084)	1.025 (0.961, 1.094)	0.961 (0.899, 1.027)
**Age**					
41−64 years (n=44,856)	1.087 (1.015, 1.165)	1.082 (1.010, 1.160)	1.031 (0.987, 1.077)	1.028 (0.952, 1.110)	0.954 (0.881, 1.032)
≥65 years (n=15,725)	1.023 (0.926, 1.131)	1.059 (0.956, 1.173)	1.061 (0.991, 1.135)	1.018 (0.903, 1.148)	0.974 (0.861, 1.102)
p-interaction	0.477	0.952	0.738	0.917	0.775
**Sex**					
Males (n=20,282)	1.066 (0.978, 1.163)	1.111 (1.017, 1.213)	1.071 (1.007, 1.139)	1.009 (0.910, 1.119)	0.995 (0.896, 1.104)
Females (n=40,299)	1.072 (0.994, 1.156)	1.047 (0.970, 1.129)	1.029 (0.983, 1.078)	1.047 (0.963, 1.138)	0.928 (0.850, 1.012)
p-interaction	0.838	0.496	0.632	0.523	0.217
**Body mass index (BMI)**					
<25 kg/m^2 (n=40,993)	1.055 (0.978, 1.138)	1.076 (0.996, 1.162)	1.039 (0.988, 1.091)	1.007 (0.924, 1.097)	0.986 (0.903, 1.077)
≥25 kg/m^2 (n=19,588)	1.099 (1.009, 1.198)	1.087 (0.998, 1.184)	1.059 (1.002, 1.118)	1.057 (0.958, 1.166)	0.919 (0.830, 1.017)
p-interaction	0.434	0.505	0.647	0.491	0.279
**Smoking status**					
Non-smoker (n=45,647)	1.070 (0.999, 1.147)	1.056 (0.986, 1.133)	1.022 (0.978, 1.068)	1.039 (0.962, 1.122)	0.933 (0.861, 1.010)
Ex-smoker (n=10,252)	1.011 (0.890, 1.149)	1.089 (0.956, 1.241)	1.071 (0.977, 1.173)	0.928 (0.794, 1.085)	1.064 (0.911, 1.241)
Current smoker (n=4682)	1.129 (0.958, 1.330)	1.138 (0.966, 1.339)	1.156 (1.040, 1.286)	1.119 (0.924, 1.356)	0.965 (0.794, 1.172)
p-interaction	0.612	0.952	0.038	0.092	0.244
**Drinking status**					
Non-drinker (n=31,544)	1.062 (0.981, 1.149)	1.079 (0.995, 1.170)	1.035 (0.984, 1.088)	1.008 (0.921, 1.105)	0.969 (0.882, 1.064)
Ex-drinker (n=4118)	0.970 (0.790, 1.190)	1.038 (0.846, 1.273)	1.037 (0.911, 1.181)	0.969 (0.760, 1.236)	1.163 (0.908, 1.490)
Current drinker (n=24,919)	1.098 (1.004, 1.200)	1.086 (0.994, 1.186)	1.064 (1.002, 1.130)	1.059 (0.959, 1.170)	0.917 (0.828, 1.016)
p-interaction	0.528	0.683	0.586	0.824	0.599
**Regular exercise**					
Yes (n=35,617)	1.039 (0.956, 1.129)	1.073 (0.989, 1.164)	1.000 (0.951, 1.052)	0.978 (0.893, 1.071)	0.984 (0.896, 1.081)
No (n=24,964)	1.079 (0.997, 1.166)	1.077 (0.994, 1.168)	1.100 (1.042, 1.161)	1.066 (0.972, 1.170)	0.947 (0.861, 1.042)
p-interaction	0.756	0.946	0.048	0.532	0.742
**Diabetes**					
Yes (n=8161)	1.033 (0.907, 1.177)	1.075 (0.944, 1.224)	1.052 (0.967, 1.144)	1.068 (0.917, 1.244)	0.936 (0.799, 1.095)
No (n=52,420)	1.068 (1.002, 1.138)	1.062 (0.996, 1.131)	1.035 (0.993, 1.078)	1.017 (0.947, 1.092)	0.964 (0.895, 1.037)
p-interaction	0.602	0.946	0.595	0.625	0.981
**Hypertension**					
Yes (n=20,982)	1.150 (1.052, 1.256)	1.086 (0.996, 1.183)	1.062 (1.003, 1.124)	1.125 (1.020, 1.242)	0.837 (0.755, 0.928)
No (n=39,599)	0.996 (0.924, 1.073)	1.038 (0.962, 1.121)	1.022 (0.973, 1.073)	0.958 (0.878, 1.044)	1.060 (0.972, 1.158)
p-interaction	0.008	0.207	0.448	0.033	0.003
**Dyslipidemia**					
Yes (n=25,092)	1.084 (0.996, 1.180)	1.076 (0.989, 1.171)	1.052 (0.996, 1.111)	1.060 (0.963, 1.166)	0.935 (0.846, 1.033)
No (n=35,489)	1.039 (0.962, 1.122)	1.049 (0.971, 1.133)	1.027 (0.977, 1.080)	0.997 (0.913, 1.089)	0.980 (0.896, 1.072)
p-interaction	0.451	0.630	0.718	0.406	0.520

Table S5. *Cont.*

	OR (95% CI)				
	**PM_{10}**	**$PM_{2.5}$**	**SO_2**	**NO_2**	**O_3**
**Cardio- and cerebrovascular disease**					
Yes (n=3390)	1.094 (0.884, 1.354)	0.963 (0.776, 1.195)	1.333 (1.167, 1.524)	1.392 (1.084, 1.788)	0.799 (0.613, 1.041)
No (n=57,191)	1.056 (0.995, 1.120)	1.068 (1.007, 1.133)	1.019 (0.981, 1.059)	1.005 (0.939, 1.074)	0.972 (0.908, 1.042)
p-interaction	0.587	0.576	0.002	0.018	0.159

PM_{10}: particulate matter with aerodynamic diameter < 10 μm; $PM_{2.5}$: particulate matter with aerodynamic diameter < 2.5 μm; SO_2: sulfur dioxide; NO_2: nitrogen dioxide; O_3: ozone; OR: odds ratio.

Estimates are reported as odds ratios and 95% confidence intervals of low-grade inflammation (hs-CRP levels > 3 mg/L) per IQR increase in 2-year average ambient air pollution exposure (corresponding IQR of each pollutant: 5.95 μg/m^3 for PM_{10}, 3.70 μg/m^3 for $PM_{2.5}$, 1.80 ppb for SO_2, 12.54 ppb for NO_2, and 4.52 ppb for O_3).

Adjusted for age, sex, BMI, smoking status, drinking status, regular exercise, occupation, education, marital status, residential area, year, season, and weekday of examination, medical history of diseases (diabetes, hypertension, dyslipidemia, cardio- and cerebrovascular disease, and cancer), and meteorological factors (2-day moving average of temperature and relative humidity). Age, sex, BMI, smoking status, drinking status, regular exercise, and medical histories were not adjusted in the stratified analysis.

Table S6. Summary statistics of the short-term residential air pollution exposure (3-day average).

Pollutant (unit)	Mean ± SD	Max	Q3	Median	Q1	Min	IQR
PM_{10} (μg/m^3)	43.44 ± 15.13	193.13	51.73	41.25	32.75	10.25	18.98
$PM_{2.5}$ (μg/m^3)	23.11 ± 9.28	125.13	28.17	21.63	16.49	2.54	11.68
SO_2 (ppb)	4.81 ± 2.92	35.40	5.75	4.07	3.00	0.13	2.75
NO_2 (ppb)	21.67 ± 10.77	77.03	28.18	19.87	13.47	0.28	14.71
O_3 (ppb)	26.92 ± 10.60	76.46	34.61	25.95	18.58	2.32	16.03

Max: maximum; Min: minimum; PM_{10}: particulate matter with aerodynamic diameter < 10 μm; $PM_{2.5}$: particulate matter with aerodynamic diameter < 2.5 μm; SO_2: sulfur dioxide; NO_2: nitrogen dioxide; O_3: ozone; SD: standard deviation; Q1, Q3: first and third quartile, respectively; IQR: interquartile range.

Table S7. Spearman correlation coefficients of short- and long-term residential air pollution exposure and meteorological factors.

	Pollutant (unit)	Short-term measures					Long-term measures					Meteorological factors	
		**PM_{10}**	**$PM_{2.5}$**	**SO_2**	**NO_2**	**O_3**	**PM_{10}**	**$PM_{2.5}$**	**SO_2**	**NO_2**	**O_3**	**Temperature**	**Humidity**
Short-term measures	PM_{10} (μg/m^3)	1.00	0.91*	0.49*	0.48*	0.12*	0.18*	0.12*	0.08*	0.12*	−0.15*	−0.24*	−0.29*
(3-day average)	$PM_{2.5}$ (μg/m^3)		1.00	0.38*	0.35*	0.10*	0.10*	0.19*	−0.02*	−0.04*	−0.04*	−0.19*	−0.12*
	SO_2 (ppb)			1.00	0.61*	−0.08*	0.09*	−0.08*	0.70*	0.40*	−0.14*	−0.20*	−0.29*
	NO_2 (ppb)				1.00	−0.37*	0.34*	−0.12*	0.47*	0.71*	−0.54*	−0.30*	−0.33*
	O_3 (ppb)					1.00	−0.10*	0.03*	−0.06*	−0.16*	0.11*	0.51*	−0.04*
Long-term measures	PM_{10} (μg/m^3)						1.00	0.59*	0.17*	0.50*	−0.66*	0.00	−0.03*
(2-year average)	$PM_{2.5}$ (μg/m^3)							1.00	−0.10*	−0.13*	−0.09*	0.00	0.13*
	SO_2 (ppb)								1.00	0.62*	−0.22*	0.02*	−0.16*
	NO_2 (ppb)									1.00	−0.78*	0.05*	−0.24*
	O_3 (ppb)										1.00	−0.04*	0.14*
Meteorological factors	Temperature (℃)											1.00	0.31*
(2-day average)	Humidity (%)												1.00

PM_{10}: particulate matter with aerodynamic diameter < 10 μm; $PM_{2.5}$: particulate matter with aerodynamic diameter < 2.5 μm; SO_2: sulfur dioxide; NO_2: nitrogen dioxide; O_3: ozone; SD: standard deviation; Q1, Q3: first and third quartile, respectively; IQR: interquartile range; * $p < 0.001$.

Table S8. Sensitivity analysis on extended model including short-term exposure measures as further covariates to the main model.

Pollutant	Adjustment of short-term exposure	% change (95% CI)[a]	OR (95% CI)[b]
PM_{10}	Main model[c]	6.377 (4.549, 8.238)	1.112 (1.011, 1.223)
	+ Short-term PM_{10} (without interaction)[d]	6.016 (4.161, 7.904)	1.109 (1.006, 1.222)
	+ Short-term PM_{10} (with interaction)[e]	10.418 (5.204, 15.890)	1.107 (1.004, 1.221)
$PM_{2.5}$	Main model[c]	10.270 (7.094, 13.541)	1.218 (1.044, 1.421)
	+ Short-term $PM_{2.5}$ (without interaction)[d]	7.022 (3.983, 10.150)	1.208 (1.032, 1.414)
	+ Short-term $PM_{2.5}$ (with interaction)[e]	8.675 (1.869, 15.935)	1.204 (1.029, 1.410)
SO_2	Main model[c]	30.769 (18.030, 44.882)	1.946 (1.114, 3.397)
	+ Short-term SO_2 (without interaction)[d]	23.005 (6.364, 42.248)	1.782 (0.809, 3.923)
	+ Short-term SO_2 (with interaction)[e]	39.686 (15.684, 68.667)[f]	1.948 (0.877, 4.326)[f]
NO_2	Main model[c]	5.224 (3.325, 7.159)	1.040 (0.940, 1.150)
	+ Short-term NO_2 (without interaction)[d]	4.678 (1.980, 7.447)	1.028 (0.889, 1.189)
	+ Short-term NO_2 (with interaction)[e]	11.703 (6.796, 16.836)[f]	1.035 (0.896, 1.196)[f]
O_3	Main model[c]	−16.130 (−20.561, −11.452)	0.835 (0.618, 1.129)
	+ Short-term O_3 (without interaction)[d]	−14.373 (−19.199, −9.259)	0.786 (0.569, 1.085)
	+ Short-term O_3 (with interaction)[e]	−1.914 (−14.218, 12.154)[f]	0.786 (0.569, 1.086)

PM_{10}, $PM_{2.5}$: particulate matter with aerodynamic diameter < 10 μm and < 2.5 μm, respectively; SO_2: sulfur dioxide; NO_2: nitrogen dioxide; O_3: ozone; Hs-CRP: high sensitivity C-reactive protein.

[a] Estimates are presented as percentage changes with 95% confidence intervals in hs-CRP levels per 1 unit increase in 2-year average ambient air pollution exposure (PM_{10}, $PM_{2.5}$, SO_2, NO_2, and O_3: 10 μg/m^3).

[b] Estimates are presented as odds ratios and 95% confidence intervals of low-grade inflammation (hs-CRP levels > 3 mg/L) per 1 unit increase in 2-year average ambient air pollution exposure (PM_{10}, $PM_{2.5}$, SO_2, NO_2, and O_3: 10 μg/m^3).

[c] Adjusted for age, sex, body mass index, smoking status, drinking status, regular exercise, occupation, education, marital status, residential area, year, season, and weekday of examination, medical history of diseases (diabetes, hypertension, dyslipidemia, cardio- and cardiovascular disease, and cancer), and meteorological factors (2-day moving average of temperature and relative humidity).

[d] Adjusted for the covariates in the main model as well as for short-term exposure (3-day moving average) of each pollutant.

[e] Adjusted for the covariates in the main model as well as for short-term exposure (3-day moving average) and the interaction term between long- and short-term exposure of each pollutant.

[f] Interaction term between short- and long-term exposure of each pollutant was statistically significant ($p < 0.10$).

Table S9. Sensitivity analysis on extended model including co-pollutants as further covariates to the main model.

Pollutant	Two-pollutant model	% change (95% CI)[a]	OR (95% CI)[b]
PM_{10}	Main model[c]	6.377 (4.549, 8.238)	1.112 (1.011, 1.223)
	+ $PM_{2.5}$[d]	5.691 (3.390, 8.043)	1.051 (0.929, 1.188)
	+ SO_2[d]	5.359 (3.414, 7.340)	1.077 (0.972, 1.194)
	+ NO_2[d]	5.298 (2.996, 7.651)	1.143 (1.014, 1.288)
	+ O_3[d]	4.500 (2.020, 7.039)	1.136 (0.997, 1.294)
$PM_{2.5}$	Main model[c]	10.270 (7.094, 13.541)	1.218 (1.044, 1.421)
	+ PM_{10}[d]	1.729 (-1.859, 5.449)	1.158 (0.950, 1.413)
	+ SO_2[d]	6.169 (3.141, 9.286)	1.182 (1.009, 1.384)
	+ NO_2[d]	8.340 (5.308, 11.460)	1.226 (1.049, 1.432)
	+ O_3[d]	6.973 (3.984, 10.047)	1.211 (1.037, 1.415)
SO_2	Main model[c]	30.769 (18.030, 44.882)	1.946 (1.114, 3.397)
	+ PM_{10}[d]	16.807 (4.629, 30.403)	1.651 (0.903, 3.018)
	+ $PM_{2.5}$[d]	24.943 (12.510, 38.750)	1.716 (0.971, 3.031)
	+ NO_2[d]	20.984 (8.420, 35.004)	1.968 (1.081, 3.582)
	+ O_3[d]	24.607 (12.316, 38.244)	1.871 (1.061, 3.298)
NO_2	Main model[c]	5.224 (3.325, 7.159)	1.040 (0.940, 1.150)
	+ PM_{10}[d]	1.743 (-0.593, 4.134)	0.954 (0.840, 1.083)
	+ $PM_{2.5}$[d]	5.689 (3.774, 7.639)	1.051 (0.949, 1.164)
	+ SO_2[d]	3.965 (1.958, 6.011)	0.994 (0.892, 1.109)
O_3	Main model[c]	−16.130 (−20.561, −11.452)	0.835 (0.618, 1.129)
	+ PM_{10}[d]	−7.742 (−14.423, −0.540)	1.101 (0.728, 1.667)
	+ $PM_{2.5}$[d]	−15.461 (−19.935, −10.737)	0.858 (0.632, 1.164)
	+ SO_2[d]	−14.523 (−19.098, −9.689)	0.886 (0.653, 1.203)

PM_{10}, $PM_{2.5}$: particulate matter with aerodynamic diameter < 10 μm and < 2.5 μm, respectively; SO_2: sulfur dioxide; NO_2: nitrogen dioxide; O_3: ozone; Hs-CRP: high sensitivity C-reactive protein.

[a] Estimates are presented as percentage changes with 95% confidence intervals in hs-CRP levels per 1 unit increase in 2-year average ambient air pollution exposure (PM_{10}, $PM_{2.5}$, SO_2, NO_2, and O_3: 10 μg/m^3).

[b] Estimates are presented as odds ratios and 95% confidence intervals of low-grade inflammation (hs-CRP levels > 3 mg/L) per 1 unit increase in 2-year average ambient air pollution exposure (PM_{10}, $PM_{2.5}$, SO_2, NO_2, and O_3: 10 μg/m^3).

[c] Adjusted for age, sex, body mass index, smoking status, drinking status, regular exercise, occupation, education, marital status, residential area, year, season, and weekday of examination, medical history of diseases (diabetes, hypertension, dyslipidemia, cardio- and cardiovascular disease, and cancer), and meteorological factors (2-day moving average of temperature and relative humidity).

[d] Adjusted for the covariates in the main model as well as for the air pollutants shown above.

Table S10. Sensitivity analysis on participants with the same place of residence.

	% change (95% CI)[a]					OR (95% CI)[b]				
	**PM_{10}**	**$PM_{2.5}$**	**SO_2**	**NO_2**	**O_3**	**PM_{10}**	**$PM_{2.5}$**	**SO_2**	**NO_2**	**O_3**
Study population (n=60,581)	6.377 (4.549, 8.238)	10.270 (7.094, 13.541)	30.769 (18.030, 44.882)	5.224 (3.325, 7.159)	−16.130 (−20.561, −11.452)	1.112 (1.011, 1.223)	1.218 (1.044, 1.421)	1.946 (1.114, 3.397)	1.040 (0.940, 1.150)	0.835 (0.618, 1.129)
Participants who did not change their residence between baseline and follow-up (n=48,451)	6.619 (4.590, 8.687)	7.916 (4.531, 11.411)	30.374 (16.278, 46.179)	5.431 (3.302, 7.603)	−17.033 (−21.921, −11.840)	1.135 (1.022, 1.261)	1.140 (0.959, 1.354)	2.367 (1.279, 4.381)	1.107 (0.990, 1.239)	0.713 (0.510, 0.998)

PM_{10}, $PM_{2.5}$: particulate matter with aerodynamic diameter < 10 μm and < 2.5 μm, respectively; SO_2: sulfur dioxide; NO_2: nitrogen dioxide; O_3: ozone; Hs-CRP: high sensitivity C-reactive protein.

[a] Estimates are presented as percentage changes with 95% confidence intervals in hs-CRP levels per 1 unit increase in 2-year average ambient air pollution exposure (PM_{10}, $PM_{2.5}$, SO_2, NO_2, and O_3: 10 μg/m^3).

[b] Estimates are presented as odds ratios and 95% confidence intervals of low-grade inflammation (hs-CRP levels > 3 mg/L) per 1 unit increase in 2-year average ambient air pollution exposure (PM_{10}, $PM_{2.5}$, SO_2, NO_2, and O_3: 10 μg/m^3).

Adjusted for age, sex, body mass index, smoking status, drinking status, regular exercise, occupation, education, marital status, residential area, year, season, and weekday of examination, medical history of diseases (diabetes, hypertension, dyslipidemia, cardio- and cardiovascular disease, and cancer), and meteorological factors (2-day moving average of temperature and relative humidity).

Table S11. Associations between other inflammatory and metabolic biomarkers and long-term exposure to air pollutants.

	**% change (95% CI)**			
	**White blood cells (10^9 units/L)**	**Fibrinogen (mg/dL)**	**Fasting blood glucose (mg/dL)**	**Triglycerides (mg/dL)**
**n[a]**	60,236	60,206	59,437	60,579
**PM_{10}**	0.766 (0.478, 1.055)	0.692 (0.492, 0.892)	0.212 (0.089, 0.336)	1.372 (0.873, 1.874)
**$PM_{2.5}$**	−0.018 (−0.310, 0.275)	0.545 (0.343, 0.748)	0.411 (0.284, 0.538)	1.691 (1.172, 2.212)
**SO_2**	1.016 (0.827, 1.206)	0.707 (0.576, 0.838)	0.031 (−0.050, 0.112)	0.559 (0.234, 0.885)
**NO_2**	1.646 (1.318, 1.975)	0.755 (0.530, 0.981)	0.052 (−0.087, 0.192)	1.207 (0.643, 1.773)
**O_3**	−1.347 (−1.674, −1.018)	−0.538 (−0.767, −0.308)	−0.106 (−0.249, 0.037)	−1.386 (−1.950, −0.818)

PM_{10}, $PM_{2.5}$: particulate matter with aerodynamic diameter < 10 μm and < 2.5 μm, respectively; SO_2: sulfur dioxide; NO_2: nitrogen dioxide; O_3: ozone; IQR: interquartile range; Q1, Q2, Q3, Q4: first, second, third, and fourth quartile, respectively.

[a] To minimize analytical laboratory errors, the extreme values (outside the mean ± 3 standard deviation after log transformation) were excluded (0.57%, 0.60%, 1.89%, and 0.003% of the observations for white blood cells, fibrinogen, fasting blood glucose, and triglycerides, respectively) before analysis.

The models are adjusted for age, sex, BMI, smoking status, drinking status, regular exercise, occupation, education, marital status, residential area, year, season, and weekday of examination, medical history of diseases (diabetes, hypertension, dyslipidemia, cardio- and cerebrovascular disease, and cancer), and meteorological factors (2-day moving average of temperature and relative humidity).

Estimates are presented as percentage changes with 95% confidence intervals for each marker per IQR increase in 2-year average ambient air pollution exposure (corresponding IQR of each pollutant: 5.95 $\mu g/m^3$ for PM_{10}, 3.70 $\mu g/m^3$ for $PM_{2.5}$, 1.80 ppb for SO_2, 12.54 ppb for NO_2, and 4.52 ppb for O_3).

Table S12. General characteristics of the study participants stratified by smoking status.

Characteristics	Non-smokers (n=45,647)	Ex-smokers (n=10,252)	Current smokers (n=4682)	*p*-value[a]
Age [years (mean ± SD)]	58.4 ± 8.0[c]	61.0 ± 8.4[b]	56.5 ± 8.1[b,c]	<0.001
Age in categories [n (%)]				
41–64 years	34,644 (75.9)	6401 (62.4)	3811 (81.4)	<0.001
≥65 years	11,003 (24.1)	3851 (37.6)	871 (18.6)	
Sex [n (%)]				
Males	6440 (14.1)	9669 (94.3)	4173 (89.1)	<0.001
Females	39,207 (85.9)	583 (5.7)	509 (10.9)	
Body mass index [kg/m^2 (mean ± SD)]	23.7 ± 2.9[c]	24.5 ± 2.8[b]	24.1 ± 2.9[b,c]	<0.001
Body mass index in categories [n (%)]				
<25 kg/m^2	31,899 (69.9)	6118 (59.7)	2976 (63.6)	<0.001
≥25 kg/m^2	13,748 (30.1)	4134 (40.3)	1706 (36.4)	
Drinking status [n (%)]				
Non-drinker	29,061 (63.7)	1769 (17.3)	714 (15.3)	<0.001
Ex-drinker	2351 (5.2)	1474 (14.4)	293 (6.3)	
Current drinker	14,235 (31.2)	7009 (68.4)	3675 (78.5)	
Regular exercise [n (%)]				
Yes	26,796 (58.7)	6643 (64.8)	2178 (46.5)	<0.001
No	18,851 (41.3)	3609 (35.2)	2504 (53.5)	
Occupation [n (%)]				
Professional, administrative	4146 (9.1)	1536 (15.0)	657 (14.0)	<0.001
Office, sales, and service	9171 (20.1)	2879 (28.1)	1528 (32.6)	
Laborer, agricultural	5179 (11.4)	2412 (23.5)	1446 (30.9)	
Others, unemployed	27,151 (59.5)	3425 (33.4)	1051 (22.5)	
Education [n (%)]				
Less than middle school (< 9 years)	7737 (17.0)	967 (9.4)	454 (9.7)	<0.001
High school (9–12 years)	25,516 (55.9)	4906 (47.9)	2412 (51.5)	
College or more (>12 years)	12,394 (27.2)	4379 (42.7)	1816 (38.8)	
Marital status [n (%)]				
Married, cohabitating	40,008 (87.7)	9604 (93.7)	4166 (89.0)	<0.001
Single, divorced, widowed, separation, others	5639 (12.4)	648 (6.3)	516 (11.0)	
Medical history				
Diabetes [n (%)]				
Yes	5281 (11.6)	2010 (19.6)	870 (18.6)	<0.001
No	40,366 (88.4)	8242 (80.4)	3812 (81.4)	
Hypertension [n (%)]				
Yes	14,804 (32.4)	4547 (44.4)	1631 (34.8)	<0.001
No	30,843 (67.6)	5705 (55.6)	3051 (65.2)	
Dyslipidemia [n (%)]				
Yes	18,459 (40.4)	4404 (43.0)	2229 (47.6)	<0.001
No	27,188 (59.6)	5848 (57.0)	2453 (52.4)	
CCVD [n (%)]				
Yes	2092 (4.6)	999 (9.7)	299 (6.4)	<0.001
No	43,555 (95.4)	9253 (90.3)	4383 (93.6)	
Cancer [n (%)]				
Yes	2868 (6.3)	630 (6.1)	139 (3.0)	<0.001
No	42,779 (93.7)	9622 (93.9)	4543 (97.0)	
Asthma [n (%)]				
Yes	1041 (2.3)	228 (2.2)	79 (1.7)	0.032
No	44,606 (97.7)	10,024 (97.8)	4603 (98.3)	
COPD [n (%)]				
Yes	56 (0.1)	43 (0.4)	11 (0.2)	<0.001
No	45,591 (99.9)	10,209 (99.6)	4671 (99.8)	
Season [n (%)]				
Spring (March–May)	6742 (14.8)	1490 (14.5)	583 (12.5)	<0.001
Summer (June–August)	15,679 (34.4)	3515 (34.3)	1587 (33.9)	
Fall (September–November)	17,857 (39.1)	4008 (39.1)	1828 (39.0)	
Winter (December–February)	5369 (11.8)	1239 (12.1)	684 (14.6)	
Residential area [n (%)]				
Metropolitan	28,892 (63.3)	6310 (61.6)	2658 (56.8)	<0.001
Non-metropolitan	16,755 (36.7)	3942 (38.5)	2024 (43.2)	
Hs-CRP [mg/L (mean ± SD)]	0.85 ± 1.10[c]	0.96 ±1.21[b]	1.14 ±1.32[b,c]	<0.001

Values are presented as mean ± standard deviation for continuous variables and n (%) for categorical variables; CCVD: Cardio- and cerebrovascular disease; COPD: chronic obstructive pulmonary disease; Hs-CRP: high-sensitivity C-reactive protein.

[a] Subgroups of smoking status were compared using one-way analysis of variance (ANOVA) test for continuous variables and Chi-square test for categorical variables. ANOVA post hoc comparisons were conducted with Bonferroni tests and the level of significance was measured at $p < 0.05$.
[b] Statistically different from non-smokers. [c] Statistically different from ex-smokers.
